# The association study between dietary behaviors, lifestyle patterns and comorbid depressive and anxiety symptoms in Yunnan adolescents aged 12–19, Southwest China: a largescale cross-section screen and a propensity score matching analysis

**DOI:** 10.3389/fpubh.2026.1956492

**Published:** 2026-10-23

**Authors:** Yunjuan Yang, Hui Fang, Wenjuan Chen, Shujuan Huang, Xiaoyun Yang

**Affiliations:** 1 Yunnan International Joint Laboratory of Public Health and Disease Control, Kunming, Yunnan, China; 2 Public Health School, Xi'an Jiaotong University, Xi'an, China; 3 Children and Adolescents Health Research Team, Yunnan Provincial Preventive Medicine Academy (Yunnan Provincial Center for Disease Control and Prevention), Kunming, Yunnan, China; 4 Public Health School, Kunming Medical University, Kunming, Yunnan, China; 5 Department of Paediatrics, Yuxi Children's Hospital, Yuxi, China

**Keywords:** adolescent, comorbid depressive and anxiety symptoms, dietary behavior, lifestyle pattern, propensity score matching analysis, sleep insufficiency

## Abstract

**Background:**

The purpose of this study is to explore the association of dietary behaviors and other lifestyle habits with the presence of comorbid depressive and anxiety symptoms in adolescents aged from 12 to 19 years old in Yunnan Province in Southwest China.

**Methods:**

We conducted a complex, multi-stage stratified, probability sampling design in 57,031 participants from 253 multi-center of 129 counties of Yunnan in 2023. Participants with complete information on basic demographic information, dietary behaviors, lifestyle patterns (incuding sleep duration, screen time, and exercises etc.,), and who participated in the survey and assessed depressive and anxiety symptoms using standardized measures. Propensity score matching was performed to reduce imbalance in prespecified measured covariates between comparison groups. Multivariable logistic regression models were used to examine the association.

**Results:**

The detection rate of total participants in the comorbid depressive and anxiety symptoms was 12.72%. And the detection rate of females in the comorbid depressive and anxiety symptoms group (15.51%) was notably higher than that of males (9.73%). After controlling for potential confounding effects through multiple logistic regression analyses, and after PSM, sedative medication misuse (OR = 4.613, 95% CI 3.734–5.699), the use of electronic devices in dark environments at night (always using: OR = 3.538, 95% CI 3.111–4.023), computer use exceeding 2 h daily (OR = 1.719, 95% CI 1.499–1.971), insufficient sleep (OR= 1.600, 95% CI 1.456–1.757), smoking (OR = 1.469, 95% CI 1.339–1.612) and alcohol consumption (OR = 1.706, 95% CI 1.572–1.851) were positive association. Consuming breakfast (OR = 0.450, 95% CI 0.380–0.532), vegetables (OR = 0.712, 95% CI 0.587–0.864), and fruits (OR = 0.705, 95% CI 0.618–0.805), the computer use < 2 h daily (OR = 0.866, 95% CI 0.801–0.936) and watching TV < 2 h (OR = 0.771, 95% CI 0.706–0.843) were negative association.

**Conclusions:**

Healthy dietary, sleep patterns, and digital device usage should be carefully monitored as part of adolescent mental health interventions. And longitudinal, culturally responsive, and comprehensive interventions addressing individual behaviors, family dynamics, and community-level factors are urgently needed.

## 1 Introduction

Researches consistently demonstrate that depression and anxiety symptoms frequently exist as comorbidities due to common clinical manifestations, behavioral characteristics, and shared genetic and environmental etiological mechanisms (1). Depression disorder and anxiety disorder are the most common mental disorders worldwide, both of which are defined by a profound and pervasive sense of depressed mood, high levels of anxiety or fear, or loss of pleasure or interest in activities (2, 3). Depression is the leading contributor to years lived with disability, while anxiety is the sixth globally (4). The co-occurring presentation of depressive and anxiety has become a particularly significant public health concern, with adolescence being the most at-risk period for its initiation (5). A previous study confirmed half of all mental health conditions start at 14 years of age (6). Approximately 50% of depressed individuals present comorbid anxiety symptoms during adolescence (7, 8).

According to the World Health Organization (WHO), 15% of adolescents experience mental disorder (9), which can lead to the potential of causing significant long-term consequences of academic hindrance, school dropout, social dysfunction, and self-harm/suicidal thoughts. Moreover, psychopathological processes developed during childhood and adolescence are known to be preserved across the lifetime, even though the symptoms change (1, 4). It obviously showed that this comorbidity significantly amplifies clinical complexity, resulting in greater symptom severity, diagnostic complexity, and substantially impaired social functioning compared to single disorders (10). The public health burden is significant. Psychiatric disorders contribute to billions of dollars of annual healthcare use and cost (11). In children and adolescents in particular, untreated comorbid depressive and anxiety symptoms are associated with multiple adverse outcomes such as low academic performance, school refusal, poor social, functioning and elevated risk for self-injury and suicidality (12). The temporal order of occurrence of these conditions, being unfavorable (the presence of anxiety authorizing the appearance of the depression (13), indicates the importance of early detection and intervention.

Based on the neurobiological and psychosocial theory (NPT), health behavior theory (HBM) and social cognitive theory (SCT), the model stresses the bidirectional relationships among individuals, behaviors, and family support. Key constructs identified include self-efficacy, outcome expectations, and observational learning, which are of particular importance in shaping adolescents' construction and sustainment of health behaviors that have an impact on mental health. Therefore, these theories provide essential frameworks for understanding how physiological factors, lifestyle behavior factors, including dietary behaviors and general lifestyle patterns, influence mental health outcomes in adolescents.

Modifiable lifestyle behavior factors are emerging as promising targets for mental health promotion and disease prevention (14). A lack of physical activity, excessive screen time, and inadequate sleep duration have been repeatedly cited as risk factors for psychosocial problems in adolescents (15–19). Engaging in physical activity is related to less emotional problems, and sleep insufficiency is related to lack of emotional regulation ability according to research. Dietary habits and quality as well as lifestyle, are essential to the maintenance of a sound psychological life. An amount of evidence support the relationship between diet and adolescents' mental health operating through multiple interconnected mechanisms, including effects on neural development, gut-brain axis communication, inflammatory processes, and neurotransmitter availability. The healthy structure and function of the brain require the presence of certain nutrients including lipids, amino acids, vitamins, and minerals. The dietary style, which is high in sugar and saturated fat, as well as in ultra-processed foods, is a known significant risk factor for adverse mental health outcomes, such as decreased cognition and mood problems in adolescents. Therefore, given these concurrent relationships with each other and with society, it is important that prevention is evidence-based and addresses the complex etiology driving adolescents comorbid depression and anxiety symptoms (including dietary and lifestyle behaviors as modifiable risk factors contributing to symptom onset and course). And maintaining adherence to healthy dietary patterns could ameliorate symptoms of depression and anxiety.

Although the emerging evidence on the link between lifestyle behaviors and mental illness is strong, significant knowledge gaps remain on the role of population-specific and contextual factors. There is a recognized need for large prospective investigations in diverse populations, especially those without a prior history of mental illness, to strengthen the evidence for primary prevention. However, little is known about the interplay of these factors among adolescents (ages 12–19), a critical period of development, particularly in economically deprived areas where dietary and lifestyle practices may be very different from those in urban affluent areas. Additionally, few studies have focused on the concurrence of comorbid depressive and anxiety symptoms in the context of these modifiable behaviors. Moreover, Yunnan Province is situated in southwest China and shares the same border of Myanmar, Laos and Vietnam, and it is a special area for adolescent mental health research. The ethnic diversity of the population living in the region including its multiple minority groups and their frequent interaction with Han Chinese populations, will offer crucial information on cultural and social correlates of mental health. Thus, the purpose of this study is to explore the association of dietary behaviors and other lifestyle habits with the presence of comorbid depressive and anxiety symptoms in a vulnerable population (adolescents aged from 12 to 19 years old) in Yunnan Province in Southwest China, a developing area of the economy. The conclusions will serve as a scientific foundation for designing targeted early behavioral interventions that prevent serious mental health problems among the most vulnerable adolescents.

Additionally, traditional modeling techniques such as regression-based models are ill-equipped to adequately deal with multidimensional covariates and favor compositional constraints, leading to biased parameter estimates and clinically misleading inferences. In order to overcome these methodological weaknesses, the present study applies the propensity score matching (PSM) technique. PSM provides a solid, transparent analytical approach in hypothesis testing via statistically paired group by a number of baseline characteristics (20). This is acting in the tradition of randomization in observational studies (21), helping to reinforce causal nuance in inference around the impact of dietary behaviors, other lifestyle behaviors, and comorbid depressive and anxiety symptoms.

Therefore, this multi-center study aims to examine the independent and synergistic effects of dietary behaviors and lifestyle patterns (including dietary, activity, and sleep etc.) on comorbid depressive and anxiety symptoms using a PSM analysis and to construct an integrated risk model that delineates the health behavior and social cognitive mechanisms from mental health. A comprehensive understanding of how specific behavioral interactions impact comorbid depressive and anxiety symptoms will inform the development of evidence-based clinical guidelines for adolescents' mental health hygiene. Ultimately, this study also aims to contribute to more effective behavioral prevention and intervention strategies for adolescents' comorbid depressive and anxiety symptoms.

## 2 Materials and methods

### 2.1 Study design and data source

This large-scale school-based cross-sectional study used de-identified data from the 2023 Prevalence and Associated Factors of Mental Health Problems Among Children in Yunnan Province of China, which was organized and implemented by Yunnan Centers for Disease Control and Prevention in Yunnan Province of Southwest China, in 2023. The survey covered 253 primary and secondary schools in all 129 counties of 16 surveillance prefectures of Yunnan Province. The target population comprised students enrolled in grades 6–12, corresponding approximately to ages 12–19 years. The present study examined overweight/obesity, depressive symptoms, and their co-occurrence among participating students.

### 2.2 Sampling procedure

A multistage stratified cluster sampling procedure was used. At the first stage, all 129 counties or districts of 16 prefectures in Yunnan Province were included in the provincial sampling framework. Within each county or district, schools were stratified by urban/rural location and school level. At the second stage, schools were randomly chosen at the level of the selected urban and rural area according to educational development index and geographical proximity to an urban or rural center. Five schools were randomly selected in each urban city, and four schools were chosen in each rural county, which was required to cover a full range of academic settings, including one junior boarding school and one senior boarding school. 253 schools were selected using simple random sampling. At the third stage, 2 intact classes from each selected grade were selected using simple random sampling. All students enrolled in the selected classes were initially eligible to be invited.

### 2.3 Participant recruitment

Selected schools were contacted through the provincial and county education authorities. School coordinators distributed study information sheets and consent forms to parents or legal guardians before the scheduled survey date. Students and their parents or legal guardians were informed of the study purpose, procedures, confidentiality protections, and the voluntary nature of participation. And written informed consent was obtained from parents and legal guardians, and assent was obtained from participating students prior to data collection.

On the survey day, trained investigators verified the consent documentation before administering the questionnaire and conducting anthropometric measurements. Students without both forms were not assessed. Declining participation did not affect students' academic standing or access to school services.

### 2.4 Eligibility and analytical sample

Students were eligible for the field survey if they were enrolled in a selected class, were present on the survey day, and had both written parent/legal guardian consent and written student assent. For the present analysis, participants were required to have valid data for depressive and anxiety symptoms and other indispensable variables. Students who self-reported a previous clinical diagnosis of depressive and anxiety disorder by a qualified healthcare professional were excluded from the analysis (As in Figure 1).

**Figure 1:**
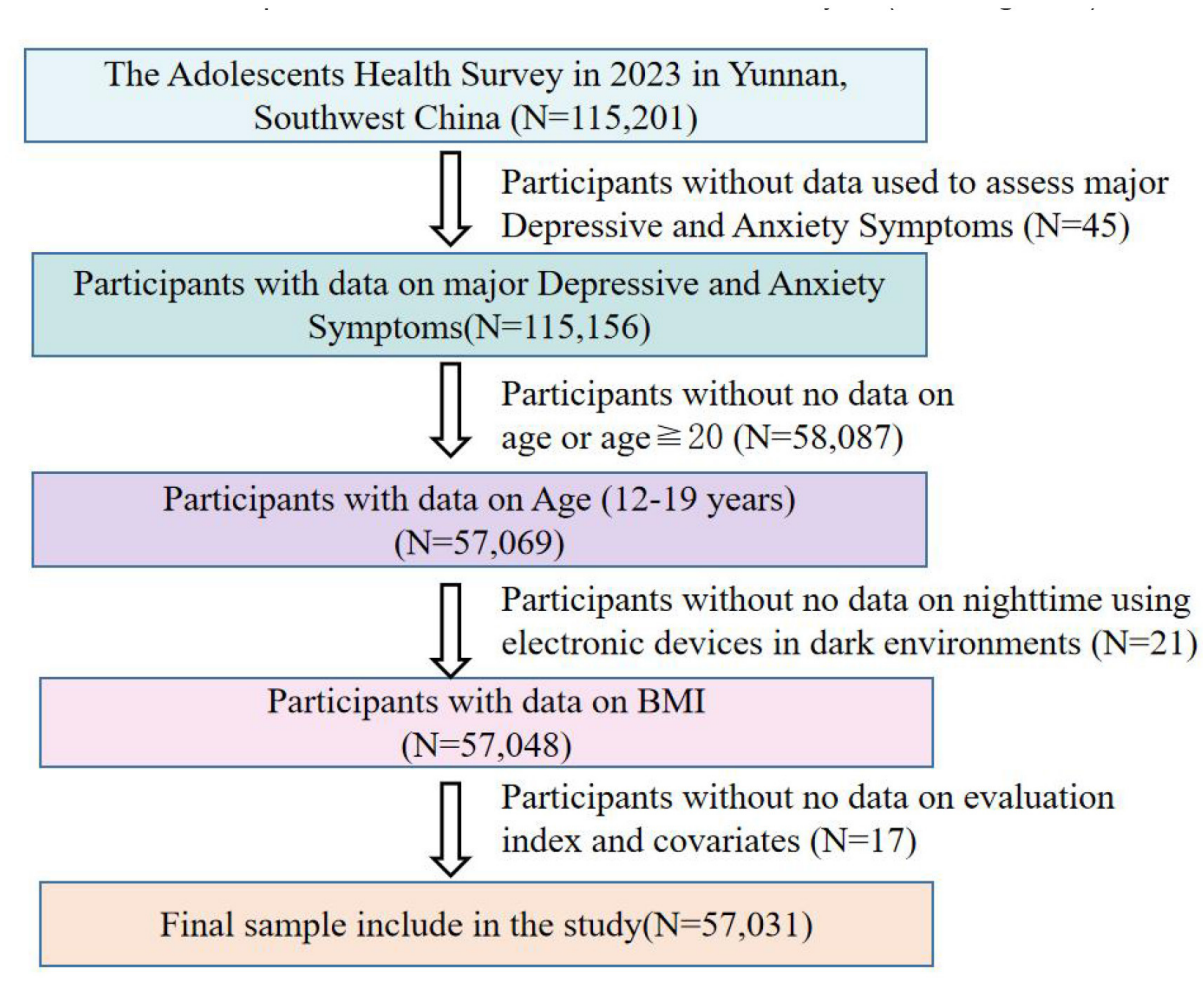
Flowchart of study participants selection.

### 2.5 Sample population

A total of 57,069 individuals initially participated in the study. After excluding records with incomplete or missing key data, the final analytic sample comprised 57,031 adolescents aged 12–19 years. This resulted in a non-response rate of 49.51%, which was taken into account in the subsequent analyses. The mean age of the included participants was 15.03 ± 1.80 years. A total of 27,528 (a percentage of 48.27%) were males and 29,503 (a percentage of 51.73%) females. In ethnic distribution, 28,884 (50.65%) were Han, 28,147 (49.35%) were ethnic groups, thus there was enough representation of both the major and minor population of Yunnan province.

With respect to educational level, 35,909 students (62.96%) were enrolled in junior high schools, whereas 21,122 students (37.04%) attended senior high schools, thereby capturing a comprehensive snapshot of adolescents at different stages of secondary education.

The rigorous approach to data collection and participant selection enhances the reliability and generalizability of the study findings within the context of adolescent health research.

### 2.6 Assessment of depressive and anxiety symptoms

The primary outcome measures of this study were comorbid depression and anxiety symptomatology.

#### 2.6.1 Depressive symptoms

Depressive symptoms were assessed using the Center for Epidemiological Studies Depression scale (CES-D) developed by Radloff L. S., National Institute of Mental Health, and included in the life style questionnaire for subjects (22). The CES-D consists of 20 items, which includes two items to measure the frequency of six major depressive symptom domains experienced in the preceding week: depressed affect, feelings of guilt or worthlessness, helplessness or hopelessness, psychomotor retardation, loss of appetite, and sleep disturbances. This instrument has been well-established as a valid and reliable screening measure for depressive symptomatology among both clinical and community samples (23).

Each item of the CES-D is scored by a four-point scale (0: rarely or none of the time; 1: some or a little of the time; 2: occasionally or a moderate amount of the time; 3: most or all of the time) which indicates the frequency of symptom in the past week. Hence, the range of the total CES-D is 0–60 with higher scores representing more severe depressive symptoms. The psychometric properties of the CES-D has been widely reported among Chinese adolescents and established to have excellent internal consistency (Cronbach α of CES-D was 0.88) (24). The internal consistency of the CES-D in the present study was Cronbach's α 0.846.

Depressive symptoms were categorized two kinds of classification: (1) No Depressive Symptoms (NDS), defined as a total CES-D score of < 16, and (2) Presence of Depressive Symptoms (DS), defined as a total CES-D score ranging from 16 to 60.

#### 2.6.2 Anxiety symptoms

The presence of anxiety symptoms was determined by using the Generalized Anxiety Disorder-7 (GAD-7) scale, a reliable and psychometrically valid measure for screening anxiety symptoms. The GAD-7 contains seven items assessing core symptoms of anxiety-related impairment over the 2-week period prior to completion (e.g., inability to control the worry, restlessness, difficulty relaxing, and irritability).

Each GAD-7 item is scored on a four-point Likert scale of 0 (“not at all”) to 3 (“nearly every day”), with a total possible score of between 0 and 21. Greater scores are indicative of more severe anxiety symptomology. The GAD-7 has shown strong reliability and validity in adolescent samples which has lead to the recommendation of this scales usefulness in the detection of clients who are in need of further evaluation for GAD. This standardization of measurement provides for the accuracy and comparability of quantifying anxiety symptoms across the study sample (25). The internal consistency of the GAD-7 in the present study was Cronbach's α 0.922.

Anxiety symptoms were categorized two kinds of classification: (1) No Anxiety Symptoms (NAS), defined as a total GAD-7 score of < 10, and (2) Presence of Anxiety Symptoms (AS), defined as a total GAD-7 score ranging from 10 to 21 (26).

#### 2.6.3 Comorbid depressive and anxiety symptoms

Participants with both depressive and anxiety mood was defined as comorbid depressive and anxiety symptoms. That is, the presence of comorbid depressive and anxiety symptoms was defined based on the simultaneous presence of elevated depressive symptoms (CES-D ≥ 16) and elevated anxiety symptoms (GAD-7 ≥ 10).

### 2.7 Dietary behaviors

Participants estimated dietary behaviors by responding to a serials of questions. (1) “Do you drink sugar-sweetened beverages in recent 7 days?”; (2) “Do you eat fried foods in recent 7 days?”; (3) “Do you eat vegetables in recent 7 days?”; (4) “Do you eat fruits in recent 7 days?”; (5) “Do you have breakfast in recent 7 days?”. The response choices consist of four categories: No, and Yes.

### 2.8 Assessment of lifestyle patterns

Lifestyle include smoking, alcohol consumption, moderate-to-vigorous activity, electronic device usage, and sleep duration.

#### 2.8.1 Current smoking status

Participants estimated current smoking status by responding to the question, “Do you smoking in recent 30 days?” The response choices consist of four categories: No, and Yes.

#### 2.8.2 Alcohol consumption status

Participants estimated aocohol consumption status by responding to the question, “Do you ever drink white wine/ red wine/ beer?” The response choices consist of four categories: No, and Yes.

#### 2.8.3 Sedative medications using status

Participants estimated sedative medications using status by responding to the question, “To date, have you ever use any sedative medications such as diazepam or triazolam?” The response choices consist of two categories: No, and Yes.

#### 2.8.4 Moderate-to-vigorous activity

Moderate-to-vigorous intensity exercise refers to any single session lasting 60 min or more that causes an increased heart rate or shortness of breath. In the study, the frequency of moderate-to-vigorous intensity exercise over the past 7 days was categorized as Low frequency (0–3 days) and High frequency (4–7 days).

#### 2.8.5 Screen usage time

According to the “Physical Activity Level Evaluation Standard for participants Aged 7 to 18”, daily screen time spent watching TV, using computers, and mobile devices of 2 h or more is classified as “high-level use,” while < 2 h is classified as “low-level use.” Participants estimated Screen usage time by responding to the question. “During the past week, how much time on an average day did you spend watching TV (including playing video games on a TV, like Xbox)?” (2) “During the past week, how much time on an average day did you spend playing on the computer?” (3) “During the past week, how much time on an average day did you spend using mobile electronic devices (including smartphones, handheld gaming consoles, tablets, etc.)?” The response choices consist of three categories: I did not watch any, < 2 h, or 2 h or more.

#### 2.8.6 Electronic device usage nighttime in dark environments

Participants estimated electronic device usage at nighttime in dark environments by responding to the question, “Do you use your devices with the lights off in the dark?” The response choices consist of four categories: Never, Sometimes, Often, and Always.

#### 2.8.7 Assessment of sleep duration

According to the Notice on Sleep Management for Primary and Secondary School Students issued by the Ministry of Education Office (27), a sleep duration of 9 h or more per day for junior high school students is considered sufficient sleep duration, and 8 h or more per day for senior high school students is sufficient sleep duration. So, this study classified the sleep duration into binary classifications: sleep insufficiency (< 8/9 h) and sufficient sleep duration (more than 8/9 h).

### 2.9 Assessment of covariates

*The body mass index (BMI)*: The BMI was calculated as field measuring body weight (kg) divided by the square of the height (m) (28).

*Family structure*: According to the family members who have lived together over the past 6 months, family structure is categorized into regular families and non-regular families (29). Regular families include nuclear families (parents and their unmarried children) and extended families (families where children live long-term with their biological parents and one or more grandparents). Non-regular families include single-parent families (families composed of one parent and their children), blended families (families where children live long-term with one biological parent and one stepparent), and skipped-generation families (families composed of grandparents and grandchildren), among others.

### 2.10 Statistical analysis

R software (version 3.6.3) was used for all statistical analysis. A two-sided *P*-value < 0.05 was interpreted as being statistically significant. Continuous data are presented in terms of means with the corresponding standard deviations (SDs) and categorical data as frequencies and percentages.

The distributions of continuous variables were assessed using histograms, Q–Q plots, and the Shapiro–Wilk test. Continuous variables with approximately normal distributions are presented as mean ± standard deviation (SD), whereas variables with non-normal distributions are summarized as median and interquartile range (IQR). Categorical variables are presented as frequencies and percentages. Between-group comparisons were performed using the independent-samples *t* test or Mann–Whitney *U* test for continuous variables, as appropriate, and the chi-square test or Fisher's exact test for categorical variables.

To reduce imbalance in prespecified measured covariates, the study used propensity score matching (PSM). Propensity scores were estimated using a logistic regression model, in which the presence of comorbid depressive and anxiety symptoms was treated as the dependent variable. Prespecified baseline covariates that were considered potential confounders based on previous literature and their potential associations with depressive/anxiety symptoms were included in the model, including age, gender, ethnic, grade, residential area, and family structure (30). Subsequently, a total of 14,512 subjects were matched for the comorbid depressive and anxiety symptoms and non-depressive and anxiety symptoms groups in a 1:1 ratio using the nearest neighbor algorithm with a caliper width of < 0.20, and without replacement for the best possible matching between the 2 groups.

The quality of matching by the algorithm was determined by absolute standardized differences (ASD), and a result of < 10% was accepted indicating sufficient balance of covariates between the two groups. Moreover, the Youden's index was used to determine the best cutoff value of distinguishing the comorbid depressive and anxiety symptoms and non-depressive and anxiety symptoms groups with receiver operating characteristic (ROC) curve. Standardized mean differences (SMDs) were also used to measure the level of covariate balance after matching.

By the use of PSM, the analysis attempted to secure comparability between groups and account for observed confounding variables, thereby increasing the internal validity of the study results (i.e., the association between comorbid depressive and anxiety symptoms and the associated outcomes). The results will report using graphs (Figure 2).

**Figure 2:**
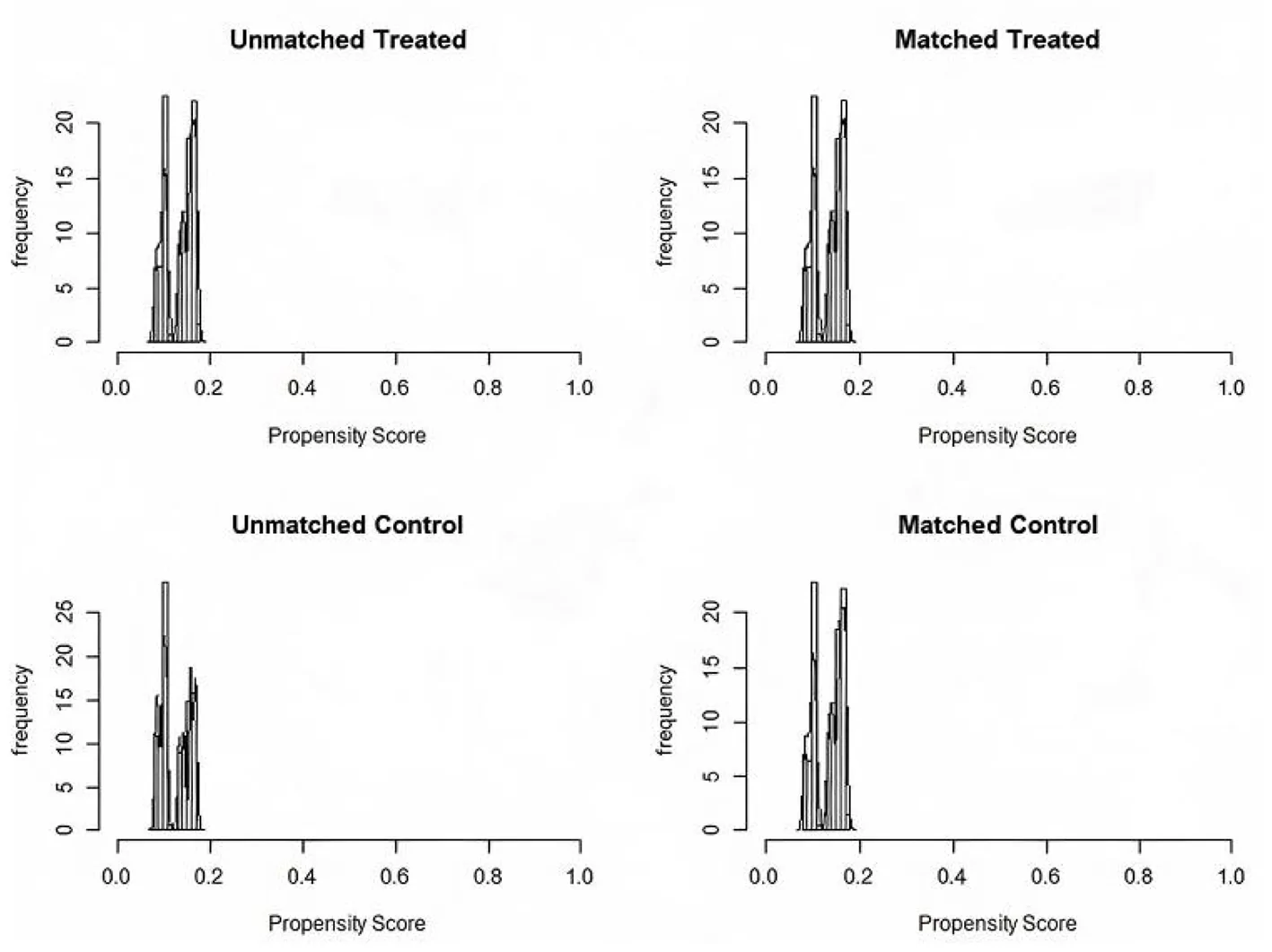
Distribution of propensity scores among participants with and without comorbid depressive and anxiety symptoms before and after propensity score matching.

For both the unmatched and propensity score-matched group, baseline differences in participant characteristics were evaluated using appropriate statistical tests. Specifically, categorical variables were compared utilizing the chi-square test, as warranted by the data distribution. Variables demonstrating statistically significant differences between the comorbid depressive and anxiety symptoms and non-comorbid depressive and anxiety symptoms groups at baseline were subsequently incorporated as covariates in further analyses (see Table 1).

**Table 1:** Characteristcs between groups before and after propensity score matching.

Characteristics	Before propensity score matching					After propensity score matching				
	comorbid depressive and anxiety symptoms group (*n* = 7,256)	Non-comorbid depressive and anxiety symptoms group (*n* = 49,775)	*Rate (%)*	*P*	*SMD*	comorbid depressive and anxiety symptoms group (*n* = 7,256)	Non-comorbid depressive and anxiety symptoms group (*n* = 7,256)	*Rate (%)*	*P*	*SMD*
Age										
12–15	5,040	34,695	12.68	0.673	0.005	5,040	4,975	50.32	0.243	−0.019
16–19	2,216	15,080	12.81			2,216	2,281	49.28		
Gender										
Male	2,679	24,849	9.73	< 0.001^**^	−0.269	2,679	4,577	36.92	1.000	0.000
Female	4,577	24,926	15.51			4,577	2,679	63.08		
Ethnic group										
Han	3,551	25,333	12.29	0.002^**^	0.039	3,551	3,634	49.42	0.168	0.023
Minority	3,705	24,442	13.16			3,705	3,622	50.57		
Grade										
Junior school	4,525	31,384	12.60	0.256	0.014	4,525	4,383	50.80	0.015^*^	−0.040
Senior school	2,731	18,391	1,2.93			2,731	2,873	4,8.73		
Area										
Urban	855	6,002	12.47	0.501	0.009	855	944	47.53	0.025^*^	0.000
Rural	6,401	43,773	12.76			6,401	6,312	50.35		
Family structure										
Regular family	2,407	18,782	11.36	< 0.001^**^	0.097	2,407	2,406	50.01	0.986	0.038
Non-regular family	4,849	30,993	13.53			4,849	4,850	49.99		

^*^P < *0.05;* ^**^P < *0.01*.

The primary outcome measure of this study was the prevalence ratio of comorbid depressive and anxiety symptoms vs. non-comorbid depressive and anxiety symptoms. To estimate the association between comorbid depressive and anxiety symptoms status and study outcomes, matched binary logistic regression models were applied to both the entire study population and the matched sample, thereby adjusting for potential confounding variables.

Furthermore, multivariate binary logistic regression analyses were conducted to calculate adjusted odds ratios (ORs) and corresponding 95% confidence intervals (CIs) for the relationships between key exposures—such as dietary behaviors and life activities—and the risk of comorbid depressive and anxiety symptoms. Separate multivariate logistic regression models were also employed to investigate the associations between specific subtypes of exposures and comorbid depressive and anxiety symptoms, thereby providing a nuanced understanding of the potential risk factors within this adolescents.

This comprehensive analytical approach ensured robust adjustment for confounders and enabled precise estimation of the associations of interest, thereby enhancing the validity and interpretability of the study findings.

## 3 Results

### 3.1 Baseline characteristics from participants

As Table 1 shown, in pre-matching data, mean age was 14.53 ± 1.82. There were 27,528 males and 29,503 females (51.73%), 28,884 Han ethnic group (50.65%), and 28,147 Minority ethnic group (49.35%). There were 35,909 Junior school students (62.96%) and 21,122 Senior school students (37.04%), respectively.

Pre-matching data revealed significant baseline characteristic differences between gender and ethnic groups (*P* < 0.001). Propensity score matching significantly improved baseline characteristic balance between the two groups. After matching, most characteristics' standardized mean differences decreased to below 0.1, indicating good matching effects. The average absolute SMD before matching was 0.285, which decreased to 0.003 after matching, showing significant balance improvement effects. There are significant differences in grade and area after matching (*P* < 0.001).

In pre-matching data, the detection rate of total participants in the comorbid depressive and anxiety symptoms was 12.72%. Among them, the detection rate of females in the comorbid depressive and anxiety symptoms group (15.51%) was notably higher than that of males (9.73%). With ethnic group' students having a higher comorbid depressive and anxiety symptoms incidence rate (13.16%) compared to Han students (12.29%; *P* < 0.01). Family structure was also an important influencing factor, with subjects from non-intact family structures having a higher comorbid depressive and anxiety symptoms incidence rate (13.63%) than those from intact families (11.36%), with statistically significant differences (*P* < 0.001, SMD = 0.097).

### 3.2 Propensity-matched analysis

PSM was performed to adjust for the heterogeneity between comorbid depressive and anxiety symptoms and non-comorbid depressive and anxiety symptoms groups. After 1:1 PSM, 100.00% of comorbid depressive and anxiety symptoms group (*n* = 7,256) and 14.58% of non-comorbid depressive and anxiety symptoms group (*n* = 7,256) were analyzed. The baseline characteristics were balanced in the matched groups (SMD < 0.2; *P* < 0.05), as presented in Table 1, Figure 2. After matching, absolute standardized differences for all measured covariates were < 10%, indicating significantly covariate balance between the two groups.

After PSM (controlling for confounding variables), many significant characteristics before matching no longer show a significant effect (e.g., gender, family structure, etc.). However, there are a characteristics that change to have significant even after matching, such as ethnic group.

### 3.3 Univariable binary logistic regression analysis

As Table 2 shown, Minority ethnic group have higher odds compared to Han ethnic group (*P* < 0.01). The odds of ethnic group decreased slightly and remained significantly to the comorbid depressive and anxiety symptoms (*P* < 0.05).

**Table 2:** Univariate analysis with binary logistic regression analyses in comorbid depressive and anxiety symptoms.

Characteristics	Before propensity score matching			After propensity score matching		
Index	*OR*	95%*CI*	*P*	*OR*	95%CI	*P*
Basic characteristics						
Age						
12–15	1.000		< 0.001^**^	1.000		0.871
16–19	1.012	0.959–1.067		0.994	0.926–1.067	
Gender						
Male	1.000		< 0.001^**^	1.000		1.000
Female	1.703	1.619–1.792		1.000	0.935–1.070	
Ethnic group						
Han	1.000		0.002^**^	1.000		0.038^*^
Minority	1.081	1.029–1.136		0.933	0.875–0.996	
Grade						
Junior school	1.000		0.256	1.000		0.918
Senior school	1.030	0.979–1.084		0.996	0.932–1.066	
Area						
Urban	1.000		0.501	1.000		0.922
Rural	0.974	0.903–1.051		0.922	0.835–−1.019	
Family structure						
Regular family	1.000		< 0.001^**^	1.000		0.659
Non-regular family	1.221	1.159–1.286		1.016	0.948–1.088	
BMI						
Normal	1.000		0.168	1.000		0.079
Overweight	1.053	0.978–1.134		1.029	0.933–1.134	
Obesity	1.126	1.028–1.233		1.150	1.017–1.301	
Daily behaviors						
Smoking						
No	1.000		< 0.001^**^	1.000		< 0.001^**^
Yes	2.086	1.977–2.201		2.428	2.244–2.626	
Alcohol drinking						
No	1.000		0.000^**^	1.000		< 0.001^**^
Yes	2.239	2.130–2.354		2.500	2.332–2.679	
Sedative medications using						
No	1.000		< 0.001^**^	1.000		< 0.001^**^
Yes	6.866	6.166–7.645		6.447	5.272–7.884	
Exercise activities						
Low frequency	1.000		< 0.001^**^	1.000		0.009
High frequency	0.855	0.809–0.903		0.909	0.845–0.976	
Sleep duration						
Sufficient sleep duration	1.000		< 0.001^**^	1.000		< 0.001^**^
Sleep insufficiency	1.752	1.635–1.876		1.646	1.511–1.794	
Dietary behaviors						
Intaking of sugar-sweetened beverages						
No	1.000		< 0.001^**^	1.000		< 0.001^**^
Yes	1.239	1.152–1.333		1.297	1.181–1.423	
Intaking of fried foods						
No	1.000		< 0.001^**^	1.000		< 0.001^**^
Yes	1.390	1.297–1.490		1.393	1.275–1.521	
Intaking of vegetables						
No	1.000		< 0.001^**^	1.000		< 0.001^**^
Yes	0.596	0.531–0.669		0.563	0.474–0.667	
Intaking of fruits						
No	1.000		< 0.001^**^	1.000		< 0.001^**^
Yes	0.638	0.587–0.692		0.600	0.533–0.675	
Breakfast consumption status						
No	1.000		< 0.001^**^	1.000		< 0.001^**^
Yes	0.369	0.336–0.406		0.340	0.291–0.397	
Vision behaviors						
Nighttime using electronic devices in dark environments						
Never	1.000		< 0.001^**^	1.000		< 0.001^**^
Occasionally	1.601	1.496–1.714		1.642	1.510–1.785	
Often	3.009	2.794–3.240		2.860	2.595–3.151	
Always	5.099	4.695–5.539		5.175	4.584–5.842	
Watching TV (hours)						
Never	1.000		< 0.001^**^	1.000		< 0.001^**^
< 2	0.663	0.625–0.704		0.664	0.614–0.718	
≧2	1.221	1.141–1.307		1.296	1.178–1.425	
Playing computer (hours)						
Never	1.000		< 0.001^**^	1.000		< 0.001^**^
< 2	0.792	0.750–0.835		0.817	0.762–0.876	
≧2	2.053	1.898–2.221		2.342	2.074–2.645	
Playing smartphone						
No	1.000		< 0.001^**^	1.000		< 0.001^**^
Yes	1.155	1.094–1.220		1.176	1.095–1.263	

^*^P < *0.05;* ^**^P < *0.01*.

The effect of dietary behaviors, lifestyle patterns remained strong, especially for smoking, alcohol drinking, exercises activities, and electronic devices usage. They showed a significant predictor and increased effect even after matching.

### 3.4 Association between dietary behaviors, lifestyle patterns, and comorbid depressive and anxiety symptoms

As Figure 3 shown, by the multivariable binary logistic regression analysis, compared with before and after matching, significant associations were found for factors such as smoking, alcohol drinking, sedative medication usage, nighttime electronic device use in dark environments, having breakfast, vegetables and fruits consumption, sufficient sleep duration, and watching TV, playing computer, smartphone usage etc.

**Figure 3:**
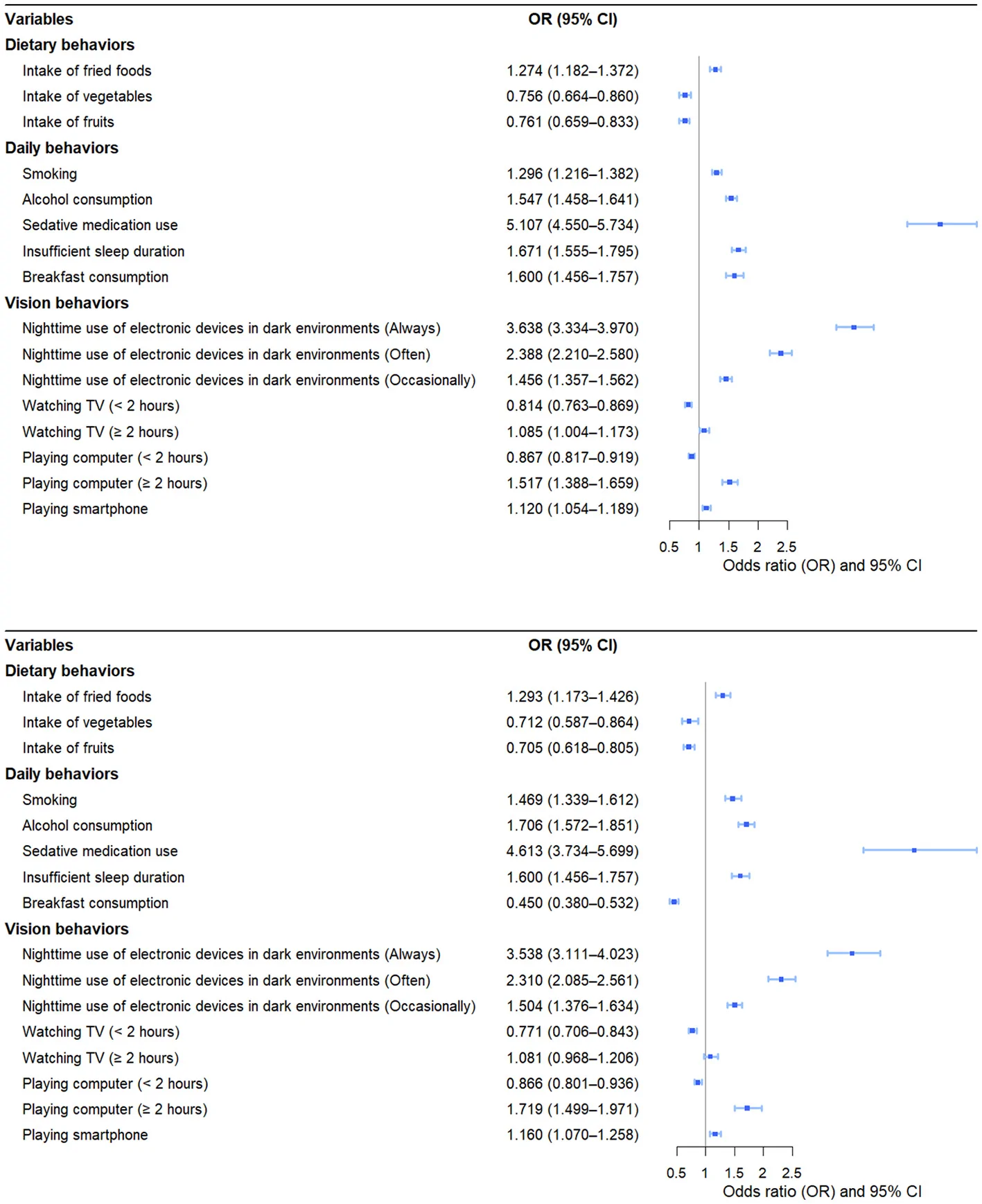
Multivariate analysis with binary logistic regression in comorbid depressive and anxiety symptoms before and after PSM. **(a)** Multivariate analysis analysis with logistic regression in comorbid depressive and anxiety symptoms (Before PSM). **(b)** Multivariate analysis with binary logistic regression in comorbid depressive and anxiety symptoms (After PSM).

These factors can divide into two types: (1) enhanced effect factors: alcohol drinking (*OR* range from 1.547 to 1.706), smoking (*OR* range from 1.296 to 1.469) and playing computer (*OR* range from), playing computer (*OR* ranging from 1.517 to 1.719) and having breakfast (*OR* ranging from 0.507 to 0.450); (2) the changes of effect was still small and has always been significant: the effects of insufficient sleep duration (*OR* ranging from 1.671 to 1.600), intaking of fried foods (*OR* ranging from 1.274 to 1.293), intaking of vegetables (*OR* ranging from 0.756 to 0.712), intaking of fruits (*OR* ranging from 0.761 to 0.705), and nighttime electronic device use in dark environments (always: *OR* ranging from 3.638 to 3.538), watching TV (*OR* ranging from 1.085 to 1.081) and smartphone usage (*OR* ranging from 1.120 to 1.160); (3) weakened effect factors: sedative medications usage (*OR* range from 5.107 to 4.613).

That is, this study's results showed the risk of comorbid depressive and anxiety symptoms in sedative medications usage, always nighttime electronic device use in dark environments, alcohol consumption, insufficient sleeping, smoking, intaking fried foods, playing computer and watching TV more than 2 h per day group (*P* < 0.01). However, there was a negative association between having breakfast, intaking vegetables and fruits, and comorbid depressive and anxiety symptoms.

## 4 Discussion

This study is the first to employ compositional methods to examine the associations between dietary behaviors, lifestyle patterns (including physical activities, smoking, alcohol drinking, and sleep duration, etc.), and comorbid depressive and anxiety symptoms among 12–19 years adolescents in Yunnan, Southwest China. We observed significant effects of dietary behaviors, unhealthy electronic device use, and other lifestyle patterns on comorbid depressive and anxiety symptoms, independent of demographic characteristics. Our findings further identified key modifiable lifestyle factors associated with mood symptoms outcomes. These findings suggest that effective interventions on modifiable dietary behaviors and unhealthy lifestyle patterns could significantly reduce the risk of comorbid depressive and anxiety symptoms.

Before PSM, our findings demonstrated that female adolescents exhibited a markedly higher prevalence of comorbid depressive and anxiety symptoms (15.51%) than males (9.73%), yielding a female-to-male ratio of 1.59:1 among Yunnan adolescents. This finding is consistent with a study from Shanghai that reported similar gender disparities in adolescent depression and anxiety comorbidity (31). These gender-specific findings also correspond with a recognized international study demonstrating higher rates of comorbid depression and anxiety symptoms in female adolescents (32, 33). The greater vulnerability among female adolescents is likely attributable to the complex interaction of biological factors (hormonal changes during puberty), psychosocial factors (increased sensitivity to interpersonal stressors), and sociocultural pressures (34). For example, during puberty hormonal changes may make them more susceptible to stress overall. The girls are under far greater stress than the boys in terms of body image and academic achievement, which disproportionately impact female adolescents (35).

Additionally, ethnic minority students showed higher rates (13.16%) than Han adolescents (12.29%). Moreover, adolescents from non-intact family structures had significantly higher rates of comorbid depressive and anxiety symptoms (13.63%) compared to those from intact families (11.36%; *P* < 0.01). This association between family structure and adolescent mental health is consistent with what was found in an extensive international study (36). Our finding of elevated comorbid symptoms in non-intact families (SMD = 0.097) is particularly concerning. These may be due to the family atmosphere and parental support. Therefore, intact family structure represents the most crucial protective factor against adolescent comorbid depressive and anxiety symptoms.

After rigorous propensity score matching to control for sociodemographic confounders, several key findings emerged that both challenged conventional assumptions and confirmed well-established modifiable risk and protective factors. Before and after PSM, the effects of modifiable factors remained robust as follows: with sedative medication misuse (*OR* from 6.866 to 6.447), use of electronic devices in dark environments at night (always using: *OR* from 5.099 to 5.175), alcohol consumption (*OR* from 2.239 to 2.500), smoking (*OR* from 2.086 to 2.428), computer use exceeding 2 h daily (*OR* from 2.053 to 2.342), insufficient sleep (*OR* from 1.752 to 1.646), and smartphone use (*OR* from 1.155 to 1.176). In contrast, eating breakfast (*OR* from 0.369 to 0.340), consuming vegetables (*OR* from 0.596 to 0.563), and eating fruits (*OR* from 0.638 to 0.600) were identified as the independent protective factors.

Notably, after controlling for potential confounding effects through multiple logistic regression analyses, and after PSM, one of the most substantial risk factors identified was taking sedative medications misuse (*OR* = 4.613, 95% *CI* 3.734–5.699) in adolescents with comorbid depressive and anxiety symptoms. This finding corroborates emerging research on prescription drug misuse among adolescents (37). Sedative medication misuse poses particular concern because it can lead to dependency and disrupt natural sleep architecture, thereby exacerbating underlying mental health vulnerabilities (38).

Furthermore, our findings revealed that smoking (*OR* = 1.469, 95% *CI* 1.339–1.612) and alcohol consumption (*OR* = 1.706, 95% *CI* 1.572–1.851) were also associated with higher odds of comorbid depressive and anxiety symptoms compared to non-smoking and non-drinking adolescents. Several neurobiological and psychosocial mechanisms have been proposed that may help contextualize these associations. Substance use may influence neurochemical systems involving in mood regulation, while adolescents experiencing psychological distress may also be more likely to initiate or maintain substance use as a coping strategy (39). In the developing brain of adolescents, chronic sedative exposure may also interfere with the natural maturation of the GABA system, leading to emotional dysregulation. Moreover, sedatives can fragment sleep architecture by both reducing Rapid Eye Movement sleep time and deep sleep stages, in which we recover from physical fatigue or emotional stress are shortened if people take them and are potentially increasing the tendency toward underlying mood disorders (40). As a result, the hypothesis of self-medication for adolescents suggests that adolescents start using substances as a way of coping with negative emotions. However, this creates a vicious cycle where substance use exacerbates underlying mental health symptoms through the effects of neuroadaptation and tolerance mechanisms.

Another important findings were the use of electronic devices in dark environments at night (always using: *OR* = 3.538, 95% *CI* 3.111–4.023), computer use exceeding 2 h daily (*OR* = 1.719, 95% *CI* 1.499–1.971), and insufficient sleep (*OR* = 1.600, 95% *CI* 1.456–1.757), all of which were independent associated with higher odds of comorbid depressive and anxiety symptoms. These findings are consistent with previous hypotheses linking nighttime electronic-device use, light exposure, circadian and sleep disruption, and poorer mental health (41). In addition, excessive device use may also coincide with reduced physical activity, shortened sleep, and fewer face-to-face social interactions.

Computer use < 2 h daily (*OR*= 0.866, 95% CI 0.801–0.936) and watching TV < 2 h (*OR*= 0.771, 95% CI 0.706–0.843) were associated with lower odds of comorbid depressive and anxiety symptoms. These findings suggest that the relationship between patterns of media use and adolescent mental health may be complex. Moderate use may coexist with healthier daily routines or different social and educational contexts, whereas excessive use may cluster with sleep disruption and other adverse lifestyle behaviors. This biphasic relationship observed with electronic device usage reflected the dual nature of digital technology's impact on adolescent development. Proper usage promotes social connection, educational opportunities, and skill development, but overuse can deprive a young person of sleep, physical activity, and face-to-face social interactions. This phenomenon suggests that structured, time-limited engagement may offer some benefits without the dependent usage patterns. This finding suggests that sleep duration and patterns of electronic device use, as well as broader lifestyle behaviors, have complex relationships with adolescents' mood outcomes and must therefore be closely monitored to determine whether they affect different populations within the digital native context. Thus, sleep patterns and digital device usage should be carefully monitored as part of adolescent mental health interventions.

Our findings also underscored the importance roles of consuming breakfast (OR = 0.450, 95% CI 0.380–0.532), vegetables (OR = 0.712, 95% CI 0.587–0.864), and fruits (OR = 0.705, 95% CI 0.618–0.805). These behaviors were similarly associated with lower odds of comorbid depressive and anxiety symptoms. Several nutritional mechanisms, including glucose metabolism, neurotransmitter synthesis, and stress regulation, have been proposed as potential explanations for associations between diet and mental health. Regular breakfast intake stabilizes blood glucose levels, prevents cortisol spikes, and mitigates mood fluctuations. Moreover, these foods provide essential nutrients for neurotransmitter synthesis, including tryptophan for serotonin production, B-vitamins for cognitive function, and tyrosine for dopamine synthesis. Recent evidence also indicates that skipping breakfast increases attentional impulsivity, mediating approximately 34.2% of the relationship between breakfast omission and depressive symptoms (42). Interestingly, traditional dietary factors such as sweets and sugary drinks did not emerge as independent risk factors, possibly reflecting Yunnan's culturally distinct dietary patterns characterized by diverse vegetables and fruits intake and lean protein sources, which support optimal brain health (43, 44).

Above all, this study highlights the multifactorial correlates of comorbid depressive and anxiety symptoms among adolescents. Sleep patterns, nighttime electronic-device use, substance use, and dietary behaviors were all associated with mental health.

Moving forward, longitudinal, culturally responsive, and comprehensive interventions addressing individual behaviors, family dynamics, and community-level factors are urgently needed. The current findings offer a roadmap for targeted preventive efforts that can substantially reduce the burden of comorbid depressive and anxiety symptoms among adolescents, thereby promoting healthier developmental trajectories and improved long-term outcomes for this vulnerable population.

### 4.1 Strengths and limitations

In this study, a number of methodological strengths served to enhance the validity, reliability, and generalizability of findings. The combination of propensity score matching and multiple logistic regressions effectively controlled for potential confounding variables, allowing for a more precise assessment of independent risk and protective factors. The large sample size, comprehensive assessment of various dietary behaviors, and lifestyle patterns indicators, and inclusion of multi-ethnic groups in Yunnan all contribute to the robustness and representativeness of the findings. These results not only shed light on region-specific adolescent mental health determinants but also enrich global understanding of cultural variations in youth psychological wellbeing. Importantly, the balanced analysis of both risk and protective factors offers a more holistic perspective than traditional deficit-oriented models.

Of course, this study also has several limitations. First, the cross-sectional design restricts causal inference; longitudinal studies are required to clarify the directionality between dietary behaviors, lifestyle patterns, and mental health outcomes. Second, several behavioral and mental health measures were based on self-reported information. In particular, sensitive behaviors such as alcohol use, cigarette smoking, and sedative use may have been underreported because of social desirability, while habitual behaviors may also have been subject to recall error. Such measurement error may have resulted in exposure misclassification and could have biased the estimated associations, although the direction and magnitude of this bias cannot be determined from present data. Third, although the large sample of adolescents from Yunnan Province provides important evidence from a culturally and ethnically diverse setting, the generalizability of these findings to adolescents in other regions of China or other countries remains uncertain because of differences in socioeconomic, cultural, and environmental contexts. Finally, detailed information on household socioeconomic status, family functioning, and ethnic-specific cultural factors was not available for the present analyses. These unmeasured factors may be associated with both behavioral exposures and mental health outcomes, and residual confounding therefore cannot be excluded. Accordingly, observed differences across ethnic or sociodemographic groups should be interpreted cautiously and should not be attributed to ethnicity or culture. Future studies incorporating more comprehensive socioeconomic, family, and culturally relevant measures are needed to better characterize these disparities.

## 5 Conclusion

This comprehensive study revealed compelling evidence that modifiable dietary behaviors and lifestyle patterns factors, particularly preventing and controlling substance misuse, keeping adequate sleep duration, regular breakfast consumption, intaking plenty of diverse vegetables and fruits, and making proper electronic device usage serve as significant protective factors against comorbid depressive and anxiety symptoms in adolescents. In addition, this study found the identification of gender and ethnic differences emphasized on carry out the targeted interventions for different groups, and family structure reflected the importance of preventing comorbid depressive and anxiety symptoms in social support system. Therefore, healthcare systems should implement comprehensive treatment and monitoring programs that track adolescent unhealthy behaviors and provide early intervention for problematic usage. Prevention programs should educate both healthcare providers and families to promote evidence-based alternatives such as cognitive-behavioral therapy for depressive, anxiety, and sleep disorders.

Further research is required to explore the specific genetic and epigenetic pathways, as well as to identify precise environmental factors that may mediate these risks. Longitudinal studies incorporating detailed environmental exposure assessments could provide more comprehensive insights into the causal pathways and potential intervention points. Additionally, exploration of whether these findings are consistent across diverse populations and settings would enhance the generalizability of our conclusions.

## Data Availability

The raw data supporting the conclusions of this article will be made available by the authors, without undue reservation.

## References

[1] Chen Z Tang Y Liu X Li W Hu Y Hu B . Edge-centric connectome-genetic markers of bridging factor to comorbidity between depression and anxiety. Nat Commun. (2024) 55:10560. doi: 10.1038/s41467-024-55008-0 39632897 PMC11618586

[2] World Health Organization. Depressive disorders [EB/OL]. Geneva: World Health Organization (2023). Available online at: https://www.who.int/news-room/fact-sheets/detail/depression (Cited March 31, 2023).

[3] World Health Organization. Anxiety disorders [EB/OL]. Geneva: World Health Organization (2023). Available online at: https://www.who.int/news-room/fact-sheets/detail/anxiety-disorders (Cited September 27, 2023).

[4] World Health Organization. Depression and other common mental disorders global health estimates. Geneva: World Health Organization (2017).

[5] Kessler RC Avenevoli S Costello EJ Georgiades K Green JG Gruber MJ . Prevalence, persistence, and Sociodemographic correlates of DSM IV disorders in the national comorbidity survey replication adolescent supplement. Arch Gen Psychiatry. (2012) 69:372–80. doi: 10.1001/archgenpsychiatry.2011.160 22147808 PMC3445020

[6] World Health Organization. World Mental Health Day 2021, key messages [EB/OL]. Geneva: World Health Organization (2021). Available online at: https://www.who.int/key-messages (Cited May 30, 2025).

[7] Long EE Young JF Hankin BL. Temporal dynamics and longitudinal co-occurrence of depression and different anxiety syndromes in youth: Evidence for reciprocal patterns in a 3-year prospective study. J Affect Disord. (2018) 234:20–7. doi: 10.1016/j.jad.2018.02.074 29522939 PMC5895498

[8] Choi JW Hong W Abela JRZ Cohen JR. Comorbid depression and anxiety symptoms in chinese adolescents: testing the explanatory power of a diathesis-anxiety model. Res Child Adolesc Psychopathol. (2021) 49:503–17. doi: 10.1007/s10802-020-00730-8 33294962

[9] World Health Organization. Improving the mental and brain health of children and adolescents[EB/OL]. Geneva: World Health Organization (2025). Available online at: https://www.who.int/activities/improving-the-mental-and-brain-health-of-children-and-adolescents (Cited May 30, 2025).

[10] Li T Zhang D Jiang T Che W Zhang Y Wan Y . A network analysis of the depression and anxiety comorbidity: a nationwide survey among Chinese adolescents during the normalization phase of COVID-19 pandemic prevention and control. BMC Psychiatry. (2025) 25:598. doi: 10.1186/s12888-025-07036-3 40598155 PMC12211814

[11] Zhang H Ding L He L Zhou R Lu W Xu T . Differential patterns of axonal loss associated with threat-related adversity in atypical depression and non-atypical depression. NeuroImage Clin. (2025) 46:103786. doi: 10.1016/j.nicl.2025.103786 40239383 PMC12020865

[12] Dai Y Shen L Zhang S Wu Z Zhang J Li Q . Trajectories and influences of depression in adolescents: a latent profile transition analysis study. Stress Health. (2025) 41:e3528. doi: 10.1002/smi.3528 39924668

[13] Xian X Niu T Zhang Y Zhou X Wang X Du X . The relationship between apearance anxiety and depression among students in a medical university in China: a serial multiple mediation model. PeerJ. (2024) 12:e17090. doi: 10.7717/peerj.17090 38563007 PMC10984188

[14] Dong Y Zhang P Zhong J Wang J Xu Y Huang H . Modifiable lifestyle factors influencing neurological and psychiatric disorders mediated by structural brain reverve: An observational and Mendelian randomization study. J Affect Disord. (2025) 372:440–50. doi: 10.1016/j.jad.2024.12.038 39672473

[15] Stubbs B. Poor physical activity as a risk factor for mental health disorders. Eur J Public Health. (2021) 31:ckab164021. doi: 10.1093/eurpub/ckab164.021

[16] van Sluijs EMF Ekelund U Crochemore-Silva I Guthold R Ha A Lubans D . Physical activity behaviours in adolescence: current evidence and opportunities for intervention. Lancet. (2021) 398:429–42. doi: 10.1016/S0140-6736(21)01259-9 34302767 PMC7612669

[17] Yang X Yang Y Yang J Ni J Li H Mu X . Short sleep duration and daytime outdoor activities effects on adolescents mental health: a stress susceptibility-recovery model analysis. J Affect Disord. (2025) 382:428–37. doi: 10.1016/j.jad.2025.04.085 40274127

[18] Kaewpradit K Ngamchaliew P Buathong N. Digital screen time usage, prevalence of excessive digital screen time, and its association with mental health, sleep quality, and academic performance among Southern University students. Front Psychiatry. (2025) 16:1535631. doi: 10.3389/fpsyt.2025.1535631 40195967 PMC11973388

[19] Yu H Xu C Lu J Li Q Li Q Zhou K . Associations between screen time and emotional and behavioral problems among children and adolescents in US, National Health Interview Survey (NHIS) 2022. J Affect Disord. (2025) 379:159–67. doi: 10.1016/j.jad.2025.03.030 40081579

[20] Kurz CF Krzywinski M Altman N. Propensity score matching. Nat Methods. (2024) 21:1770–2. doi: 10.1038/s41592-024-02405-4 39284960

[21] Fei W. Propensity score matching: should we use it in designing observational studies?. BMC Med Res Methodol. (2025) 25:25. doi: 10.1186/s12874-025-02481-w 39875810 PMC11776168

[22] Radloff LS. The use of the center for epidemiologic studies depression scale in adolescents and young adults. J Youth Adolesc. (1991) 20:149–66. doi: 10.1007/BF01537606 24265004

[23] Radloff LS. The CES-D scale: a self-report depression scale for research in the general population. Appl Psychol Meas. (1977) 1:385–401. doi: 10.1177/014662167700100306

[24] Chen Z Yang X Li X. Psychometric features of CES-D in Chinese adolescents. Chin J Clin Psychol. (2009) 17:443–5. (Chinese).

[25] Spizer RL Kroenke K Williams JBW Lowe B. A brief measure for assessing generalized anxiety disorder: the GAD-7. Arch Intern Med. (2006) 166:1092–7. doi: 10.1001/archinte.166.10.1092 16717171

[26] Han ZR Fang H Ahemaitijiang N Wang H. Generalized anxiety disorder scale (GAD-7). In:Medvedev ON Krägeloh CU Siegert RJ Singh NN, editors. Handbook of Assessment in Mindfulness Research. Cham: Springer (2024). doi: 10.1007/978-3-030-77644-2_87-1

[27] Ministry of Education General Office. Notice on further strengthening sleep management for primary and secondary school students [EB/OL] (2021).. Available online at: https://www.gov.cn/zhengce/zhengceku/2021-04/02/content_5597443.htm (Cited March 30, 2021).

[28] Luo Q Bao K Gao W Xiang Y Li M Zhang Y. Joint effects of depressive status and body mass index on the risk of incident hypertension in aging population: evidence from a nationwide population-based cohort study. BMC Psychiatry. (2023) 23:608. doi: 10.1186/s12888-023-05105-z 37598204 PMC10439533

[29] Ying M. New Family Sociology. Wuhan: Huazhong University of Science and Technology Press (2000).

[30] Andrew BY Alan Brookhart M Pearse R Raghunathan K Krishnamoorthy V. Propensity score methods in observational research: brief review and guide for authors. Br J Anaesth. (2023) 131:805–9. doi: 10.1016/j.bja.2023.06.054 37481434

[31] Wang Y Zhang X Li Y Qin H Li X. Gender differences in the prevalence, correlated factors and comorbidity of depression in adolescents: a cross-sectional study in Shanghai, China. Front Public Health. (2024) 12:1436413. doi: 10.3389/fpubh.2024.1436413 39712306 PMC11659128

[32] Riecher-Rossle A. Sex and gender differences in mental disorders. Lancet Psychiatry. (2017) 4:8–9. doi: 10.1016/S2215-0366(16)30348-0 27856397

[33] McLean CP Asnaani A Litz BT Hofmann SG. Gender differences in anxiety disorders: prevalence, course of illness, comorbidity and burden of illness. J Psychiatr Res. (2011) 45:1027–35. doi: 10.1016/j.jpsychires.2011.03.006 21439576 PMC3135672

[34] Sisk LM Gee DG. Stress and adolescence: vulnerability and opportunity during a sensitive window of development. Curr Opin Psychol. (2022) 44:286–92. doi: 10.1016/j.copsyc.2021.10.005 34818623 PMC9007828

[35] Agam R Tamir S Golan M. Gender differences in respect to self-esteem and body image as well as response to Adolescents' School-based Prevention Programs. J Psychol Clin Psychiatry. (2015) 2:00092. doi: 10.15406/jpcpy.2015.02.00092

[36] Jensen TM Xinran L. Bidirectional associations between family belonging and youth well-being over time: family structure as a moderating influence. J Adolesc Health. (2025) 0:1–6. 40946237 10.1016/j.jadohealth.2025.07.005

[37] Gao T Jin M Alibudbud R Assanangkornchai S Sun Y Lu L. Etomidate misuse: a digital era threat to youth and a call for anticipatory control. Lancet Psychiatry. (2025) 12:732–3. doi: 10.1016/S2215-0366(25)00267-6 40967727

[38] Grant JE Lust K Chamberlain SR. Sedative/Tranquilizer misuse is associated with alcohol and illicit drug problems, mental health issues, and impulsivity and compulsivity in university students. J Addict Med. (2019) 14:199–206. doi: 10.1097/ADM.0000000000000556 31403522 PMC7272224

[39] Sarah D Jun Z. Substance abuse and neurotransmission. Adv Pharmacol. (2022) 93:403–41. doi: 10.1016/bs.apha.2021.10.007 35341573 PMC9759822

[40] Hutka P Krivosova M Muchova Z Tonhajzerova I Hamrakova A Mlyncekova Z . Association of sleep architecture and physiology with depressive disorder and antidepressants treatment. Int J Mol Sci. (2021) 22:1333. doi: 10.3390/ijms22031333 33572767 PMC7866255

[41] Blume C Garbazza C Spitschan M. Effects of light on human circadian rhythms, sleep and mood. Somnologie. (2019) 23:147–56. doi: 10.1007/s11818-019-00215-x 31534436 PMC6751071

[42] Wong SMY Choi O Suen YN Hui CLM Lee EHM Chan SKW . Breakfast skipping and depressive symptoms in an epidemiological youth sample in Hong Kong: the mediating role of reduced attentional control. Front Psychiatry. (2025) 16:1574119. doi: 10.3389/fpsyt.2025.1574119 40485925 PMC12141854

[43] Sun Y Zuo T Cheung CP Gu W Wan Y Zhang F . Population-level configurations of gut mycobiome across 6 ethnicities in urban and rural China. Gastroenterology. (2021) 160:272–86.e11. doi: 10.1053/j.gastro.2020.09.014 32956679

[44] Charisis S Yannakoulia M Scarmeas N. Diets to promote healthy brain ageing. Nat Rev Neurol. (2025) 21:5–16. doi: 10.1038/s41582-024-01036-9 39572782

